# Unraveling the psychological and behavioral pathways connecting classroom instruction to mathematics achievement: insights from TALIS Shanghai data

**DOI:** 10.3389/fpsyg.2026.1831480

**Published:** 2026-08-26

**Authors:** Weitong Liu, Xinwei Chang, Yuxuan Yan, Hui Zhang

**Affiliations:** 1 School of International Education, Shandong University, Jinan, Shandong, China; 2 Institute for Advanced Studies in Education, Shandong University, Jinan, Shandong, China; 3 School of Education, University of Glasgow, Glasgow, United Kingdom

**Keywords:** academic achievement, classroom interaction, classroom management, cognitive engagement, subject-specific efficacy, teacher emotional support

## Abstract

**Background:**

Understanding the complex classroom pathways that underlie student learning outcomes is vital for pedagogical improvement. While prior scholarship highlights isolated instructional variables via survey methods, empirical insights regarding the simultaneous behavioral and psychological pathways in real classroom settings remain scarce.

**Methods:**

Using secondary data from the 2018 Teaching and Learning International Survey (TALIS) Global Teaching Insights (GTI) video study in Shanghai, China (*N* = 85 mathematics teachers; *N* = 2,613 students), this study applies Structural Equation Modeling (SEM) to examine the concurrent associations among classroom management, teacher emotional support, and mathematics achievement via the distinct mediating mechanisms of classroom interaction (quantity/quality), cognitive engagement, and subject-specific self-efficacy.

**Results:**

The results reveal a dual effect of classroom management: while it improves the quality of interactions, it simultaneously reduces the frequency of interaction, thereby weakening students’ efficacy and achievement. In contrast, emotional support from teachers enhances both the frequency and quality of classroom interactions, ultimately promoting cognitive engagement and academic performance. Moreover, interaction quantity and quality exert comparable influences on outcomes, with cognitive engagement and subject-specific efficacy functioning as key mediators.

**Conclusion:**

These findings shed light on the psychological and behavioral dynamics underlying classroom improvement, highlight the value of emotionally supportive and interaction-balanced teaching practices, and provide empirical support for both social-cognitive and constructivist theories of learning.

## Introduction

Exploring which factors can explain variations in student learning outcomes is crucial for improving classroom teaching. Educational researchers typically identify and investigate different levels of factors affecting student learning outcomes to provide practical recommendations for enhancing education (Gao and Lv, 2024). Creemers and Kyriakides (2015) noted that, over the past 40 years, researchers from various countries have suggested that, compared to the school level, the classroom level has a more significant impact on student achievement (Creemers and Kyriakides, 2015; Kyriakides et al., 2020). Moreover, differences in classroom teaching quality can largely be attributed to teachers’ teaching behaviors (what teachers do and how they interact with students) rather than their characteristics (Carbonneau et al., 2024).

Learning outcomes are the most direct and compelling evidence of teaching effectiveness (Kyriakides et al., 2013). In previous empirical studies exploring which teaching factors influence (and how they influence) student learning outcomes, researchers have predominantly used questionnaire data to verify or improve teaching theories or to focus on specific issues within classroom teaching (Maulana et al., 2016; Chen, 2023). However, there is a lack of exploration of the internal mechanisms through which various classroom teaching factors affect learning outcomes in real-life settings, making it challenging to systematically provide feasible paths for improving classroom teaching. In 2020, based on TALIS, OECD released the “Global Teaching Insights Video Study” (GTI) project report (OECD, 2020), which added a video study component, making it possible to gain an in-depth and systematic understanding of how teachers’ teaching practices affect student learning outcomes. Building on this dataset, the present study aims to investigate how psychological and behavioral factors jointly shape classroom teaching effectiveness and student achievement.

## Literature review

### Classroom management and social–emotional support: foundational factors in effective teaching

Effective classroom management is widely recognized as a cornerstone of successful pedagogy, exerting a profound influence on both teaching effectiveness and student learning outcomes (Pianta et al., 2008). It involves a broad range of teacher-initiated actions designed to establish and sustain a classroom environment that fosters students’ academic, behavioral, and social–emotional development (Evertson and Weinstein, 2006; Poulou et al., 2022). This includes strategies for managing student misbehavior, implementing discipline and rules, and providing constructive guidance to support student engagement and self-regulation (Evertson and Weinstein, 2006; Kunter et al., 2007; Ding et al., 2008; Chen and Lu, 2022). As Brophy (2006) succinctly defines, classroom management encompasses “all actions taken to create and maintain a learning environment conducive to successful instruction” (pp. 17–43).

Classroom management is a pivotal aspect of effective teaching that plays a crucial role in fostering an optimal learning environment. It is through effective classroom management that educators can establish a structured and predictable setting, which is vital for student engagement and academic advancement (Evertson and Weinstein, 2006). Effective classroom management creates a structured and supportive environment that promotes students’ learning motivation by providing clear rules and encouraging active involvement (HD and Darmawan, 2023). This fosters mutual respect, reduces disruptive behaviors, and increases on-task engagement, optimizing instructional time. Additionally, adept classroom management techniques contribute to a positive and inclusive classroom climate that nurtures social and emotional learning, encouraging students to actively participate, take academic risks, and engage in collaborative learning activities, which are essential for cognitive development and knowledge construction (Pianta and Hamre, 2009).

A growing body of research highlights the significance of classroom management in shaping the learning environment and enhancing student achievement (Evertson and Weinstein, 2013). Previous studies have shown that teachers’ actions in the classroom have a greater impact on student academic performance than school-level policies concerning curriculum, assessment, staff collaboration, and community involvement (Marzano and Marzano, 2003). The empirical efficacy of classroom management strategies is further substantiated by Korpershoek et al. (2016), whose meta-analysis of 54 intervention studies found that classroom management strategies have small yet significant positive effects on students’ academic, behavioral, and social–emotional outcomes. However, recent studies suggest that the effectiveness of classroom management strategies may vary across cultural and regional contexts. For instance, Chen and Lu (2022) found that while classroom management positively influenced students’ academic emotions and achievement in England, it did not consistently enhance students’ enjoyment or academic performance in Hong Kong. These findings indicate that the impact of classroom management is not universal and may be influenced by contextual factors such as educational policies, cultural norms, and student expectations. Even in the current digital age, classroom management remains equally important. With the help of digital information and communication technology, it can guide students to actively participate in classroom activities and improve their academic performance (Mahmoud and Bawaneh, 2025).

Building upon the critical role of classroom management in fostering an effective learning environment, social–emotional support is essential for enhancing students’ well-being, engagement, and overall academic success. The Classroom Assessment Scoring System (CLASS), developed by Pianta, La Paro, and colleagues, underscores the significance of a nurturing classroom climate in addressing students’ emotional and social needs. Teachers play a pivotal role in fostering positive relationships characterized by respect, encouragement, and warmth, which contribute to students’ emotional security and motivation to learn. Research suggests that a supportive classroom atmosphere not only enhances student engagement and academic performance but also promotes resilience, reduces anxiety, and fosters a growth mindset (Ambrose et al., 2010). The positive emotions of teachers can have a positive impact on students’ academic performance, either through direct transmission effects or through their teaching behaviors (Frenzel et al., 2021). Therefore, integrating social–emotional support into daily instructional practices is crucial for creating an inclusive and empowering learning environment that strengthens students’ self-efficacy, sense of belonging, and long-term academic development.

A substantial body of research underscores the pivotal role of social–emotional support in fostering students’ academic success by enhancing their self-efficacy, motivation, and engagement. Empirical studies have consistently demonstrated that teacher emotional support significantly influences students’ social–emotional well-being (Farmer et al., 2011; Li et al., 2021), which in turn enhances their behavioral, cognitive, and emotional engagement in learning (Allen et al., 2013; LoCasale-Crouch et al., 2018; Pianta et al., 2021; Zhou et al., 2023). If teachers communicate with students respecting their peers, this may encourage students’ feeling of safety and discourage anxiety (Ryan and Patrick, 2001). Furthermore, research highlights that teachers’ emotional expressions and enthusiasm for teaching are critical factors shaping the quality of classroom interactions, fostering a responsive and supportive learning environment that benefits both students’ motivation and overall well-being (Lee et al., 2016; Burić and Moè, 2020; Poulou et al., 2022). When students perceive strong social–emotional support from teachers, it not only improves the quality of classroom interactions and relationships (Garner et al., 2019) but also initiates a reinforcing cycle: as students develop greater confidence in their abilities, they become more invested in learning, ultimately leading to higher academic achievement. Additionally, studies have linked perceived teacher affective support to key academic outcomes, including student achievement, learning behaviors, and self-efficacy beliefs (Ma et al., 2021; Pishghadam et al., 2023; Zhao and Yang, 2022; Kaya et al., 2022). Collectively, these findings underscore that teacher-provided social–emotional support is not only instrumental in strengthening student-teacher relationships but also serves as a critical mechanism for fostering academic resilience and long-term success.

### Classroom interaction and subject matter quality: key drivers of teaching effectiveness

Current educational consensus emphasizes that teaching transcends mere information transmission, serving instead to scaffold autonomous learning and collaborative knowledge construction (Wells and Arauz, 2006). Empirical meta-analyses (Bransford et al., 2000; Vermunt and Verloop, 1999) confirm that learning quality is strongly correlated with students’ engagement in exploratory activities, which require teachers to design dynamic interactions through activity organization and time management. Among these, the quality of teacher-student interactions and classroom discourse plays a pivotal role in shaping students’ deep conceptual understanding (Alexander, 2006; Howes, 2010; Mercer and Littleton, 2007; Nystrand, 1997). Recent research further emphasizes that high-quality teacher-student interactions enhance academic performance and relationship-building through the mediating effect of effective communication (Dahmani et al., 2024), reinforcing their centrality in knowledge construction.

Recently, numerous scholars in learning sciences underscore the mechanisms by which teacher-student interactions foster cognitive participation. For instance, Linn and Eylon (2011) suggested that teacher-student interactions can elicit students’ existing knowledge. By encouraging students to articulate their thoughts, their ideas become visible to both teachers and peers, aiding teachers in providing scaffolding based on students’ prior knowledge. Furthermore, Lilly et al. (2023) has proved through empirical research that the student-centered interactive models (such as inquiry-based learning and peer assessment) can significantly enhance the learning achievements and problem-solving abilities in STEM subjects.

And the study by Košir and Tement (2014) echoes the significance of the quality of teacher-student interactions in enhancing cognitive engagement. Their research indicates that a reciprocal relationship exists between teacher acceptance and student achievement, suggesting that a nurturing teacher-student dynamic is instrumental in fostering a deeper level of student involvement in their learning process. In this context, interactions that are characterized by collaborative learning and deep dialogues are particularly effective. Jurado-Castro et al. (2023) notes that real-time classroom interactive competition seems to help students learn through an environment of communication and interaction that encourages feedback and reinforcement between teachers and students, and between students themselves with their peers. Such engagements encourage students to actively participate, reflect on their understandings, and work collectively towards a shared learning objective.

To further amplify cognitive participation, teachers can act as facilitators who guide students to delve deeper into topics, consider diverse viewpoints, and provide feedback that fosters growth. This scaffolding not only enhances the classroom discourse but also supports students in developing their analytical and argumentative skills. The supportive and interactive learning environment, as suggested by Košir and Tement (2014), is crucial for transforming students from passive recipients of knowledge to active constructors of understanding. It is within this enriched educational ecosystem that the true potential of teacher-student interactions for enhancing cognitive engagement is realized.

High-quality subject matter serves as a critical foundation for fostering meaningful classroom interactions by enhancing students’ motivation and cognitive engagement. Research indicates that well-structured, relevant, and intellectually challenging content stimulates internal curiosity and sustains learners’ attention, creating fertile ground for active participation. For instance, Linnenbrink-Garcia et al. (2010) demonstrated that the quality of academic content significantly stimulates students’ situational interest and self-efficacy, which in turn drives their willingness to engage in discussions, ask questions, and collaborate with peers. When content is aligned with learners’ prior knowledge, it reduces cognitive overload (Sweller, 2010), allowing students to allocate mental resources toward higher interactions such as critical analysis and problem-solving. Raccanello et al. (2022) analyzed the moderating effect of the quality of course content on the effectiveness of group cooperative learning, and found that when the subject matter was highly structured and profound, the interaction efficiency and knowledge transfer ability of students were significantly enhanced. This alignment between content quality and cognitive readiness ensures that classroom dialogues are not merely superficial exchanges but opportunities for deep exploration.

### Cognitive engagement and subject-specific efficacy: psychological mechanisms of learning outcomes

Cognitive engagement, defined as the extent to which students are invested in and actively process learning material, is a critical determinant of academic success (Fredricks et al., 2004). Research highlights that both the quality of classroom interaction and social–emotional support significantly enhance students’ cognitive engagement. High-quality classroom interactions, characterized by effective instructional strategies that challenge students’ thinking, are strongly associated with increased cognitive engagement (Pianta et al., 2012; Hattie, 2009). Such interactions not only promote critical thinking but also create an environment conducive to deeper learning. Equally important is the role of social–emotional support from teachers. When educators demonstrate empathy, provide constructive feedback, and foster positive relationships, they significantly enhance students’ motivation and willingness to engage cognitively (Roorda et al., 2011; Granziera et al., 2022). Tao et al. (2022) meta-analysis even found that the perceived emotional support has a greater impact on students’ academic performance than autonomy or academic support. This finding is further supported by recent research showing that psychological well-being—including self-acceptance, purpose in life, and environmental mastery—acts as a buffer against negative cognitive-emotional influences and strengthens resilience, especially among STEM students (Gonta and Tripon, 2025). Research by Jennings and Greenberg (2009) further underscores the importance of a supportive classroom climate in promoting cognitive engagement. The interaction between high-quality classroom interactions and robust social–emotional support creates a synergistic effect that amplifies cognitive involvement. For instance, a teacher who effectively combines engaging content with emotional support can cultivate an environment that maximizes students’ cognitive engagement (Skinner and Belmont, 1993). This integrated approach not only addresses immediate learning needs but also fosters long-term academic success by developing students’ inherent motivation and self-regulated learning skills (Zimmerman, 2002).

Bandura (1977) proposed that self-efficacy, is the ability of students to solve problems in the learning process and the prediction of their own ability to achieve, and it is the degree of confidence in their own ability. Self-efficacy can influence whether an individual attempts to cope with challenging or threatening situations, even more so, it being said that it plays a central role in the exercise of individual initiative (Bandura, 1977, 1991). The subsequent research applies the self-efficacy theory to subject learning, namely subject-specific efficacy (Al-Abyadh and Abdel Azeem, 2022). A substantive body of research from the field of educational psychology has shown that students’ beliefs about their capabilities are important indicators of how well students learn and perform (Usher, 2015). Through previous research, both classroom interaction and subject content quality have a significant impact on subject efficacy. We might expect that observing a teacher perform cognitive skill operations through classroom interaction could increase students’ learning self-efficacy, because modeling implicitly conveys that they possess the capabilities to succeed and will do so if they perform the same sequence of actions (Schunk, 1985).

### The present study

Building upon the existing research on classroom management, social–emotional support, classroom interaction, and subject matter quality, the present study aims to empirically examine the mechanisms through which these factors influence student learning outcomes. While prior studies have extensively discussed the theoretical significance of these components, few have quantitatively analyzed their interrelationships using large-scale empirical data. By leveraging the 2018 TALIS dataset from Shanghai, China, this study constructs a structural equation model (SEM) to systematically investigate the influence pathways of classroom teaching on student achievement.

This study focuses on three key dimensions: (1) the role of classroom management in shaping classroom interactions and student engagement, (2) the impact of teacher emotional support on fostering a supportive learning environment, and (3) the mediating effects of cognitive engagement and subject-specific efficacy on learning outcomes. Prior literature suggests that classroom management has both positive and negative implications—it can enhance interaction quality while potentially limiting interaction quantity. Additionally, teacher emotional support has been found to positively influence both interaction quality and quantity, which in turn shape students’ cognitive engagement and overall performance. However, the extent to which these relationships hold in a real-world educational setting remains underexplored.

The study proposes the following research questions:

RQ1: How does classroom management influence student learning outcomes?

RQ2: What is the impact of teachers’ social–emotional support on student academic performance?

RQ3: How do the quantity and quality of classroom interactions affect student learning outcomes?

RQ4: To what extent do cognitive engagement and subject-specific efficacy mediate the relationship between classroom teaching practices and student learning outcomes?

By addressing these questions, this study seeks to provide a data-driven understanding of effective classroom teaching strategies, offering practical implications for educators and policymakers. The findings will contribute to a more nuanced comprehension of the complex interactions within classroom teaching and inform evidence-based interventions aimed at improving student achievement.

### Conceptual framework

Based on existing literature, this study identifies seven key dimensions in the classroom teaching process: classroom management, social–emotional support, quantity of classroom interaction, quality of classroom interaction, quality of subject matter, cognitive engagement, and subject-specific efficacy. These dimensions serve as indicators of the teaching process in the classroom. The outcome indicator reflecting teaching quality is students’ learning outcomes, specifically mathematics achievement as measured by TALIS.

**Figure 1:**
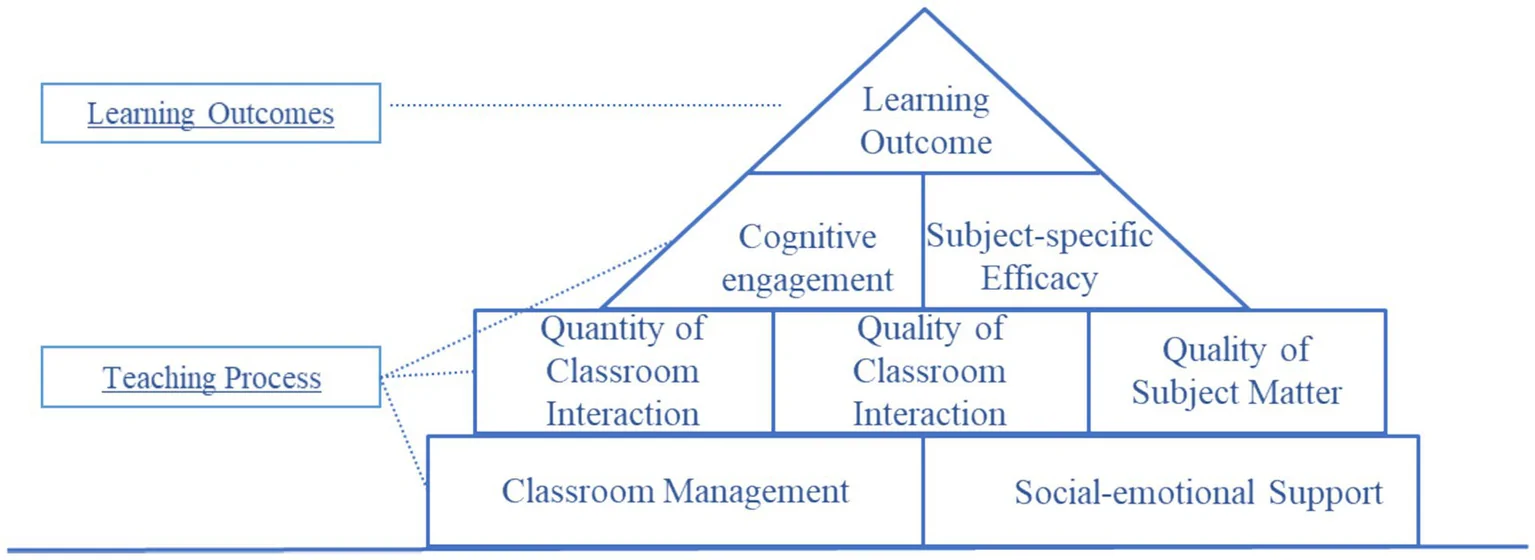
Framework of classroom teaching’s influence on learning outcomes.

Figure 1 illustrates a four-tier pyramid model of teaching process and performance indicators. The base consists of classroom management and social–emotional support, representing classroom climate indicators unrelated to subject knowledge. The third tier includes the quantity and quality of classroom interaction, and quality of subject matter, which are related to knowledge transmission and sharing. The second tier focuses on cognitive engagement and subject-specific efficacy. The apex represents the ultimate learning outcomes. This study constructs a hypothetical model of classroom teaching based on a literature review and uses the classroom video analysis database from the GTI cross-national study for quantitative data. The hypothesis is tested using SEM analysis. The aim is to balance teacher-student interaction, knowledge content construction, and classroom climate creation, reflecting the high complexity of classroom teaching.

## Data and method

### International assessment context: TALIS and TIMSS

This study is grounded in two internationally recognized large-scale assessment programs: TALIS and the Trends in International Mathematics and Science Study (TIMSS). While both are designed to inform education policy and practice through cross-national data, they differ in research focus, data types, measurement approaches, and target populations (Mullis et al., 2020).

TIMSS, implemented by the International Association for the Evaluation of Educational Achievement (IEA), measures students’ academic achievement in mathematics and science at Grades 4 and 8 through standardized cognitive tests. TIMSS also collects contextual information via background questionnaires administered to students, teachers, and school principals (Mullis et al., 2020). While effective at mapping the relationships between students’ cognitive-social factors and academic outcomes, TIMSS falls short in capturing the impact of real-time instructional practices. Specifically, it relies on generalized, retrospective surveys prone to memory and social desirability biases, and lacks standardized, direct video observations of actual classroom teaching.

TALIS, conducted by OECD, investigates the learning environments and working conditions of teachers and school leaders through standardized self-report questionnaires. It captures information on teachers’ instructional practices, beliefs, professional development experiences, and school climate across participating countries and economies (OECD, 2020). A specialized component of TALIS is the GTI study, which expands upon the original survey design by incorporating systematic video-based classroom observations alongside teacher and student questionnaires (OECD, 2020). GTI provides rich, multidimensional data that enables the examination of instructional quality through both subjective and observational measures. This multi-method blueprint allows our study to accurately break down the hidden behavioral and psychological mechanisms connecting instructional actions to performance metrics.

To summarize, TALIS-GTI and TIMSS assessments are complementary in scope and methodology. While TALIS-GTI emphasizes how teachers teach, TIMSS focuses on what students learn. Their integration allows for a comprehensive investigation of the link between classroom instruction and student learning outcomes. Both programs adhere to rigorous international standards for sampling, instrument design, and data quality, ensuring the comparability and reliability of their findings for cross-national education research (OECD, 2019; IEA, 2021).

### The GTI study and data

Building upon the influential TIMSS Video Studies (1995 and 1999) and other existing research, the OECD launched the GTI study in 2016 to examine teaching practices across different cultural contexts. This initiative aimed to identify key aspects of teaching that influence student learning and development. The city of Shanghai participated in the study, known for its high-performing students in the 2009 and 2012 cycles of the Programme for International Student Assessment (PISA) (Zhu and Kaiser, 2022).

Originally known as the TALIS Video Study, the project was later renamed GTI study. It aimed to evaluate classroom teaching effectiveness through direct classroom observations, expanding upon the foundation established by TALIS. Drawing on national teaching standards and international research on teaching quality (e.g., Praetorius and Charalambous, 2018), the GTI study developed a conceptual framework comprising six key domains of teaching practice. Classroom Management encompasses the structures and strategies teachers employ to maintain an orderly learning environment, ensuring high levels of student attention in mathematics while minimizing disruptions. Social–Emotional Support refers to the creation of a positive learning climate characterized by mutual respect, encouragement, and intellectual risk-taking. Quality of Subject Matter involves the clarity of learning goals, the use of mathematical representations, and the articulation of conceptual connections and patterns. Students’ Cognitive Engagement captures the depth of students’ involvement in learning, including the level of cognitive demand, their understanding of rationales, opportunities for practice and metacognition. Assessment and Response to Student Understanding: examines how teachers elicit student thinking, provide feedback on reasoning, and adapt instruction accordingly. Classroom Discourse focuses on patterns of student participation, analyzing who speaks, the nature of questions posed, and the types of explanations provided. The latter four subdomains were collectively categorized under the broader instructional domain within the GTI study. Further details on the development of these domains can be found in the technical reports (Bell et al., 2018; OECD, 2020).

To ensure cross-national comparability, quadratic equations were selected as the focal mathematics topic, as this content was covered in all participating regions. The GTI study involved approximately 700 mathematics teachers and 17,500 students from various countries and regions, including Chile (three cities), Colombia, the United Kingdom (England), Germany (seven federal states), Japan (three regions), Spain (Madrid), Mexico, and China (Shanghai).

### Our secondary study: data sources and analytical methods

This study focuses on Shanghai’s dataset, which consists of a stratified random sample of 85 junior high school mathematics teachers and 2,613 students, along with their corresponding classroom teaching data and personal learning-related information. Since 2009, Shanghai has actively participated in international large-scale assessments such as PISA and TALIS, and the same time demonstrated high levels of student achievement and teacher quality (Huang, 2020). These results have brought international attention to Shanghai’s basic education system.

To ensure a rigorous quantitative analysis, the eight instructional and psychological variables in our structural model are operationally defined and measured in strict alignment with the TALIS-GTI framework, integrating expert video observations with student self-report questionnaires. Detailed definitions for each variable are presented below. Their components and corresponding item codes in the TALIS-GTI framework are provided in Appendix A, and descriptive statistics and variable correlations in Appendix B.

1. Classroom Management (Expert Video Rating): Operationally defined as the structural mechanisms and teacher-led behaviors aimed at maximizing instructional time and maintaining an orderly environment. It is measured via standardized rater scores across three components: Routines (smoothness of transitions), Monitoring (teacher awareness of classroom dynamics), and Disruptions (low frequency of student misbehavior).
2. Social–Emotional Support (Expert Video Rating): Operationally defined as the cultivation of a nurturing, safe, and low-anxiety classroom climate. It is indicated by expert scores on Respect (positive teacher-student tone, valuing student dignity) and Encouragement & Warmth (teacher praise, emotional support, and reassurance during problem-solving).
3. Quality of Subject Matter (Expert Video Rating): Operationally defined as the structural clarity and cognitive depth of the mathematical content presented. Indicators include the accuracy of Explicit Connections made between mathematical procedures and concepts, and the depth of Explicit Patterns & Generalizations.
4. Quality of Classroom Interaction (Expert Video Rating): Grounded in the GTI “Classroom Discourse” domain, this variable captures the cognitive depth of exchanges. It is measured by expert ratings on Questioning (the use of open-ended, deep inquiries rather than simple yes/no prompts) and Explanations (opportunities for students to justify their mathematical reasoning).
5. Quantity of Classroom Interaction (Expert Video Rating): Measures the breath and frequency of participatory opportunities. Indicators encompass the Nature of Discourse (balance of teacher-vs-student talk time), student Risk-taking in voicing unique ideas, and overall Discussing Opportunities afforded during the lesson.
6. Students’ Cognitive Engagement (Student Self-Report): Operationally defined as the psychological investment and mental effort students allocate to comprehending the subject matter. This is captured via post-unit questionnaire scales evaluating students’ self-assessed Actual Cognitive Engagement during the quadratic equations unit.
7. Subject-Specific Efficacy (Student Self-Report): Operationally defined as students’ subjective beliefs in their capability to successfully execute tasks in mathematics. This is measured via a validated questionnaire scale reflecting students’ confidence in solving quadratic equations under their current teacher’s instruction.
8. Students’ Learning Outcomes (Standardized Achievement Test): Operationally defined as students’ demonstrated academic performance in mathematics following instructional intervention (OECD, 2020; Praetorius et al., 2020a, 2020b). This is measured via standardized post-test achievement scores (POST_TEST*) assessing students’ mastery of targeted quadratic equation concepts and problem-solving abilities.

The purpose of this study is to examine the key factors influencing classroom teaching and their impact on student achievement. Using the 2018 TALIS data from Shanghai, China, we developed a structural equation model to analyze the relationships among classroom management, social–emotional support, classroom interaction, subject matter quality, student cognitive engagement, subject-specific efficacy, and learning outcomes. Drawing on theoretical and empirical insights, we conceptualized the relationships between these constructs as independent, mediating, and dependent variables. SEM was employed to simultaneously examine the theoretically specified direct and indirect associations among the observed GTI indicators within a single model (Hair et al., 2014). The model was primarily theory-driven rather than developed solely on the basis of modification indices. Model fit was assessed using indices such as the goodness of fit index (GFI), normed fit index (NFI), comparative fit index (CFI), incremental fit index (IFI), Tucker-Lewis index (TLI), and root-mean-square error of approximation (RMSEA). The structural model was evaluated by examining the relationships among observed variables and assessing the overall model fit.

Note that while Shanghai’s outstanding educational performance and well-developed instructional systems make it a valuable case for investigating the dynamics of classroom teaching, several structural and cultural characteristics set Shanghai apart from many other educational systems. Structurally, Shanghai operates under a highly centralized curriculum planning system with rigorous, high-stakes accountability mechanisms at both the school and district levels (OECD, 2016; Tan, 2017). Culturally, classrooms are deeply embedded in Confucian values, which inherently emphasize high academic standards, strong respect for teacher authority, and a traditional preference for structured, collective instruction over individualistic exploration. Consequently, variables like Classroom Management and Classroom Interaction must be interpreted within this unique context where high behavioral compliance is culturally normative, potentially limiting direct generalizability to highly decentralized or Western educational systems.

## Results

### Test of structural model

**Figure 2:**
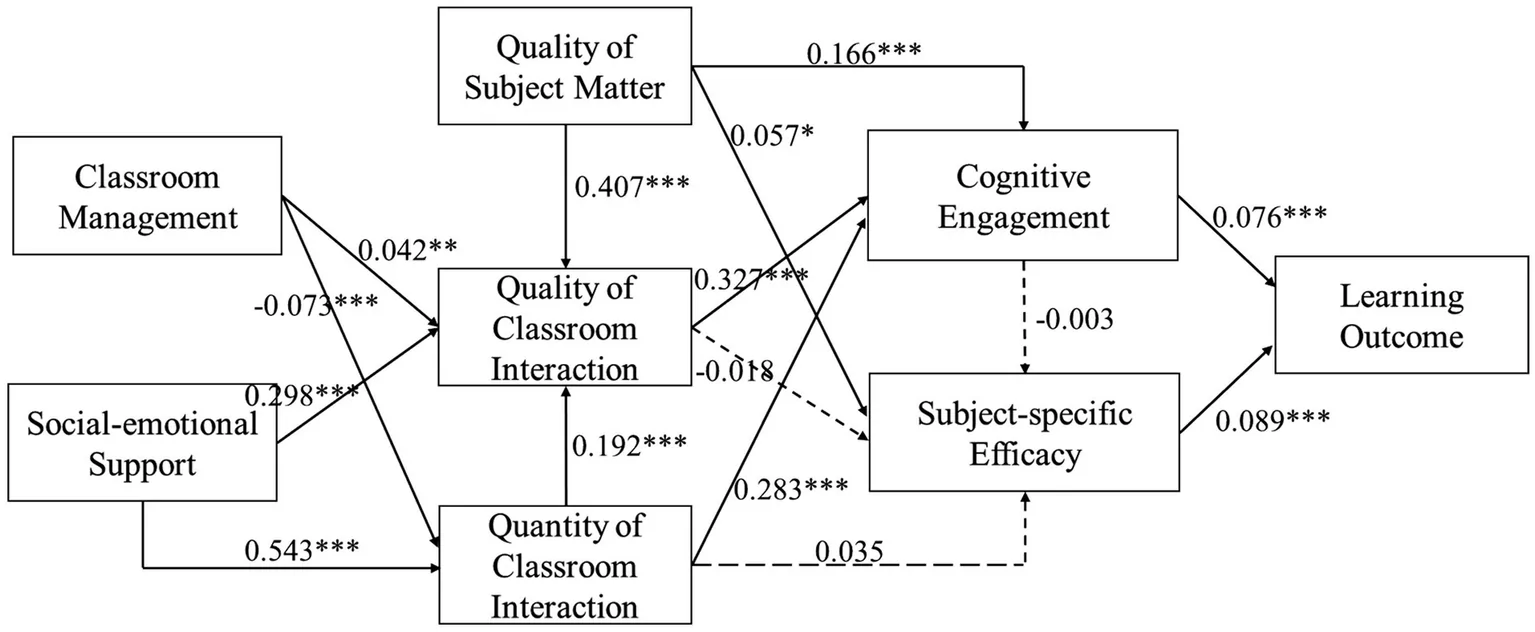
Structural equation model analysis.

The SEM approach was utilized to evaluate the proposed model examining relationships among classroom management, social–emotional support, quality of subject matter, classroom interaction (quantity and quality), cognitive engagement, subject-specific efficacy, and student learning outcomes. The overall model fit indices indicated an excellent fit to the empirical data (*χ^2^/df = 2.746; GFI = 0.998; AGFI = 0.991; PGFI = 0.194; NFI = 0.996; CFI = 0.998; IFI = 0.998; TLI = 0.991; RMSEA = 0.026*). Figure 2 provides a graphical representation of the structural model, highlighting significant paths with solid lines and non-significant paths with dashed lines.

Solid lines indicate that the path coefficients are significant, while dashed lines indicate that the path coefficients are not significant. ^⁎^*p* < 0.05, ^⁎⁎^*p* < 0.01, ^⁎⁎⁎^*p* < 0.001.

### Path coefficient significance test

The significance test of path coefficients is the primary method for examining whether the model path parameters have statistical meaning. The significance of path coefficients within the structural equation model is detailed in Table 1, demonstrating the strength and directionality of hypothesized relationships among variables. Classroom management was significantly and negatively associated with the quantity of classroom interaction (*β = −0.073, p < 0.001*), indicating that stricter management practices may reduce interaction frequency. Conversely, classroom management positively influenced the quality of classroom interaction (*β = 0.042, p < 0.01*), suggesting a structured environment could enhance interaction effectiveness. Social–emotional support exhibited significant positive associations with on both the quantity (*β = 0.543, p < 0.001*) and quality of classroom interactions (*β = 0.298, p < 0.001*), highlighting the critical role of emotional support in fostering productive student-teacher interactions. Furthermore, the quality of subject matter positively influenced the quality of classroom interaction (*β = 0.407, p < 0.001*), cognitive engagement (*β = 0.166, p < 0.001*), and subject-specific efficacy (*β = 0.057, p < 0.05*), emphasizing its comprehensive impact on various educational outcomes. The quantity of classroom interaction positively influenced the quality of classroom interaction (*β = 0.192, p < 0.001*) and cognitive engagement (*β = 0.283, p < 0.001*), though it did not significantly affect subject-specific efficacy. Additionally, the quality of classroom interaction predicted higher cognitive engagement (*β = 0.327, p < 0.001*) but did not significantly affect subject-specific efficacy. Lastly, cognitive engagement (*β = 0.076, p < 0.001*) and subject-specific efficacy (*β = 0.089, p < 0.001*) significantly and positively influenced student learning outcomes, underscoring their central mediating roles in translating instructional practices into tangible academic success.

**Table 1:** Path analysis and path coefficients of structural relationship.

Path	Standardized coefficient (*β*)	S.E.	*C.R.*	*p-value*
Classroom management → quantity of classroom interaction	−0.073	0.078	−4.441	***
Classroom management → quality of classroom interaction	0.042	0.046	3.039	**
Social–emotional Support → Quantity of Classroom Interaction	0.543	0.021	32.773	***
Social–emotional support → quality of classroom interaction	0.298	0.015	17.314	***
Quality of subject matter → quality of classroom interaction	0.407	0.014	26.672	***
Quality of subject matter → cognitive engagement	0.166	0.021	9.243	***
Quality of subject matter → subject-specific efficacy	0.057	0.391	2.333	*
Quantity of classroom interaction → quality of classroom interaction	0.192	0.012	11.463	***
Quantity of classroom interaction → cognitive engagement	0.283	0.015	16.394	***
Quantity of classroom interaction → subject-specific efficacy	0.035	0.288	1.475	0.140
Quality of classroom interaction → subject-specific efficacy	−0.018	0.490	−0.628	0.530
Cognitive engagement → subject-specific efficacy	−0.003	0.366	−0.108	0.914
Quality of classroom interaction → cognitive engagement	0.327	0.024	17.008	***
Cognitive engagement → learning outcome	0.076	1.888	3.777	***
Subject-specific efficacy → learning outcome	0.089	0.132	4.583	***

^⁎^*p* < 0.05, ^⁎⁎^*p* < 0.01, ^⁎⁎⁎^*p* < 0.001.

### Reporting of impact effect values

Table 2 displays the standardized direct, indirect, and total effects among the classroom teaching variables on student learning outcomes within the proposed structural model. The total standardized effects represent the combined impact of both direct and indirect paths, offering a comprehensive view of how each factor contributes to student achievement. Specifically, classroom management demonstrates a negative direct effect on the quantity of classroom interaction (−0.073) but positively influences the quality of interaction (0.042). Social–emotional support significantly and positively impacts both interaction quantity (0.543) and interaction quality (0.298). Moreover, the quality of subject matter exerts substantial direct positive influences on classroom interaction quality (0.407), students’ cognitive engagement (0.166), and subject-specific efficacy (0.057). Both the quantity (0.283) and quality (0.327) of classroom interactions directly enhance students’ cognitive engagement. Cognitive engagement (0.076) and subject-specific efficacy (0.089) directly promote student learning outcomes. Considering indirect effects, social–emotional support significantly impacts cognitive engagement (0.285) and learning outcomes (0.022). Similarly, the quality of subject matter has notable indirect effects on cognitive engagement (0.133) and learning outcomes (0.028). Classroom management shows minor negative indirect effects on cognitive engagement (−0.011) and learning outcomes (−0.001). The total standardized effects reveal the relative importance of each factor. Subject-specific efficacy (0.089) and cognitive engagement (0.076) have the greatest overall impacts on learning outcomes, followed by the quality of subject matter (0.028), quality of classroom interaction (0.026), quantity of classroom interaction (0.025), and social–emotional support (0.022). Classroom management exhibits an overall slight negative total effect (−0.001), emphasizing the necessity of a balanced approach between maintaining classroom order and promoting interactive learning opportunities.

**Table 2:** Standardized direct, indirect, and total paths within the structural model.

Dependent variables		Independent variables						
		Classroom management	Social–emotional support	Quality of subject matter	Quality of classroom interaction	Quantity of classroom interaction	Cognitive engagement	Subject-specific efficacy
Standardized direct effects	Quantity of classroom interaction	−0.073	0.543					
	Quality of classroom interaction	0.042	0.298	0.407	0.192			
	Cognitive engagement			0.166	0.283	0.327		
	Subject-specific efficacy			0.057				
	Learning outcome						0.076	0.089
Standardized indirect effects	Quality of classroom interaction	−0.014	0.104					
	Cognitive engagement	−0.011	0.285	0.133	0.063			
	Learning outcome	−0.001	0.022	0.028	0.026	0.025		
Standardized total effects	Quantity of classroom interaction	−0.073	0.543					
	Quality of classroom interaction	0.028	0.402	0.407	0.192			
	Cognitive engagement	−0.011	0.285	0.299	0.346	0.327		
	Subject-specific efficacy			0.057				
	Learning outcome	−0.001	0.022	0.028	0.026	0.025	0.076	0.089

## Conclusion and discussion

This study utilized SEM to investigate the associations among classroom teaching processes, psychological mediators, and student learning outcomes, drawing on TALIS data collected from Shanghai, China. The results provide empirical evidenceempirical evidence regarding these relationships and may inform refinement of instructional strategies and optimization of teaching effectiveness. By integrating the findings with existing literature and international TALIS research, this section contextualizes the implications, clarifies theoretical contributions, and offers practical recommendations for educators and policymakers.

### The paradox of classroom management: balancing order and engagement

Classroom management is recognized by the OECD as a fundamental aspect of teaching quality, significantly impacting student learning experiences. Our analysis highlights a paradoxical role of classroom management. Consistent with Pianta et al. (2012), well-structured classroom management systems provide stability necessary for effective learning yet are concurrently linked to a reduction in student-initiated discourse.^1^ This dual impact aligns with Doyle’s (2013) ecological perspective, cautioning that an overly rigid management approach can diminish students’ autonomy, particularly among adolescents. While structured classroom management positively influences the quality of classroom interactions, it simultaneously reduces interaction frequency, negatively affecting student engagement and learning outcomes. Hence, educators must strike a delicate balance between maintaining classroom order and fostering a participatory environment conducive to active student engagement. Recent innovations, such as gamified classroom management integrating elements like the Wizard of Oz, have been found effective in boosting student motivation and participation (Chen et al., 2024).

### Strengthening social–emotional support for enhanced student outcomes

As emphasized by Schonert-Reichl (2017), creating a safe, supportive, and participatory classroom environment is essential for student success. Our findings underscore social–emotional support from teachers as a particularly influential factor, surpassing traditional classroom management practices in its positive association with student outcomes. Higher levels of social–emotional support significantly enhance both the quantity and quality of classroom interactions, consequently increasing students’ cognitive engagement and academic performance. These results echo Curby et al. (2013), who found superior academic and social outcomes for preschoolers in emotionally supportive classrooms. Similarly, Rucinski et al. (2018) highlighted that emotional support improves teacher-student relationships, positively affecting students’ academic trajectories. More recently, Yang et al. (2021) found that teacher emotional support was associated with mathematics performance through students’ academic self-efficacy and behavioral engagement, further supporting the mediating pathways identified in the present study. Thus, incorporating emotional intelligence training and strategies for creating emotionally supportive classrooms into professional development programs could substantially enhance educational outcomes.

### Rethinking classroom interaction: the interplay of quantity and quality

This study underscores the equal importance of both the quantity and the quality of classroom interactions in enhancing cognitive engagement and learning outcomes. Prior research has established that interactive classrooms facilitate deep learning, with structured and meaningful dialogue playing a critical role in promoting student comprehension and knowledge construction. Specifically, high-quality and intensive interactions encourage learners to actively construct knowledge, inspire them to pursue learning objectives rigorously, and invest greater mental effort and energy into their learning process (Jung and Lee, 2018). Our findings further substantiate this perspective, emphasizing that interaction frequency alone is inadequate unless accompanied by meaningful cognitive engagement. Similarly, Prediger et al. (2024) found that only specific features of classroom interaction quality predicted students’ conceptual learning gains, suggesting that the educational value of interaction depends on its substantive content rather than interaction alone. Drawing upon Chi’s ICAP framework (2014, 2018), it is clear that cognitively active interactions, such as constructive dialogue, are strongly predictive of conceptual understanding, whereas passive interactions like rote recitation yield minimal educational benefits. Consequently, instructional strategies should simultaneously promote increased frequency and enhance the substantive depth of classroom interactions. Even within traditional teacher-led classrooms, employing basic questioning techniques can facilitate meaningful exchanges with students. Through such approaches, educators can realize the cognitively constructive and interactive engagement, ultimately advancing students’ overall learning performance.

### Mediating roles of cognitive engagement and subject-specific efficacy

SEM results reveal cognitive engagement and subject-specific efficacy as critical mediators in the relationship between instructional practices and student learning outcomes. Classroom management, social–emotional support, subject matter quality, and classroom interactions primarily map to learning outcomes through these mediators. Bandura’s (1977) social cognitive theory posits self-efficacy as a central mediator shaping human achievement, which is supported by our findings. These outcomes also resonate with constructivist learning theories, emphasizing cognitive engagement and self-efficacy as crucial to knowledge internalization processes. Bada and Olusegun advocate that effective classroom environments teach students how to learn, a principle increasingly relevant in contemporary online educational settings, where learner-content interaction predominantly determines cognitive engagement levels (Wang et al., 2022). Furthermore, in the context of artificial intelligence (AI) integration in education, higher levels of AI capability significantly boost student creativity, self-efficacy, and academic performance (Wang et al., 2023). Consequently, educators are encouraged to leverage AI-enhanced tools to further elevate student learning experiences and outcomes. Recent empirical research provides convergent yet complementary evidence that self-efficacy and engagement serve as psychological mechanisms linking teacher support with mathematics engagement and performance, although their specific roles vary across studies, functioning as mediators in some models and moderators in others (Gan and Peng, 2024; Yang et al., 2021). In the present study, the nonsignificant path from cognitive engagement to subject-specific efficacy (*β* = −0.003, *p* = 0.914) further suggests that these variables may operate as distinct rather than sequential mechanisms, possibly because of differences in educational stage and model specification.

It is critical to note that while the structural model identified several statistically significant pathways, the absolute magnitudes of certain specific effects are remarkably small. For instance, the path from classroom management to the quality of classroom interaction (*β* = 0.042, *p* < 0.01), as well as the direct impacts of cognitive engagement (*β* = 0.076, *p* < 0.001) and subject-specific efficacy (*β* = 0.089, *p* < 0.001) on standardized mathematics learning outcomes, exhibit modest explanatory power. In large-scale datasets like the TALIS-GTI study, minor behavioral variations can easily reach statistical thresholds without necessarily translating into transformative pedagogical shifts. Therefore, these small coefficients suggest that while structured environment and psychological variables are statistically valid nodes in the instructional pipeline, their standalone operational impact on final test scores is only marginal.

### Implications

From a theoretical perspective, this study contributes to the understanding of classroom dynamics by elucidating the complex pathways through which different instructional factors interact to shape learning outcomes. It extends existing frameworks by empirically demonstrating the dual nature of classroom management and the pivotal role of social–emotional support in instructional effectiveness. Practically, these findings suggest that teacher training programs should emphasize balanced classroom management strategies, equipping educators with tools to maintain order while fostering an interactive and supportive learning environment. This includes establishing flexible routines that allow for collaborative group transitions and designing co-created classroom norms that grant students autonomy during discussions, thereby eliminating the negative path from management to interaction quantity. Moreover, rontline teachers should consciously combine the quantity and quality of classroom dialogue. Rather than relying on traditional, rapid IRF (Initiation-Response-Feedback) cycles that favor rote recitation, educators should employ dialogic questioning techniques. Teachers can utilize open-ended prompts (e.g., “How did you arrive at this solution?” or “Can someone propose an alternative method to solve this quadratic equation?”) to scaffold student explanations. This ensures that an increase in interaction frequency translates effectively into high-quality cognitive engagement. Finally, given that social–emotional support exerts powerful total effects on both interaction dimensions, teachers should treat emotional security as a prerequisite for cognitive risk-taking. In practice, this means intentionally validating students’ errors as valuable learning opportunities, maintaining an encouraging and warm tone when students struggle, and actively reducing mathematics anxiety before introducing high-demand tasks.

Within the domain of educational policy and curriculum design, policymakers should systematically integrate emotional regulation and supportive climate-building into teacher development programs to establish strong foundations for productive classroom discourse. Concurrently, strategic resource allocation must prioritize curricula with built-in interaction spaces—such as exploratory tasks and conceptual prompts—that effectively balance deep cognitive engagement with flexible pacing. Ultimately, curriculum designers and educational leaders need to embed pedagogical approaches that simultaneously elevate interaction quality and sustain student engagement across learning environments.

### Limitations

While this study provides valuable insights into the psychological and behavioral dynamics of classroom improvement, several limitations should be acknowledged to contextualize the findings and guide future research.

First, the empirical data analyzed in this research were drawn exclusively from Shanghai, an exceptional educational system characterized by an intense cultural emphasis on education, a highly centralized governance framework, ongoing curriculum reforms, and consistently superior student achievement metrics (Tucker, 2011; Huang, 2020). These unique institutional and sociocultural characteristics introduce significant boundary conditions regarding the generalizability of our findings. For example, the strong positive effects of teacher emotional support and the specific trade-offs of classroom management observed here are situated within a culture that naturally highly respects teacher authority and accepts high levels of behavioral structure. In educational systems characterized by highly decentralized policies, lower stakes accountability, or individualist cultural norms, the structural paths—particularly the dynamics between classroom order, interaction frequency, and student psychological mediators—may manifest differently. Future cross-national comparative studies utilizing the full global GTI dataset are necessary to validate the invariance of our structural model across diverse cultural regimes.

Second, due to the cross-sectional nature of the TALIS 2018 dataset, this study cannot establish definitive causal relationships between classroom processes and student learning outcomes. In addition, because the 2,613 students were nested within 85 mathematics teachers/classrooms, the single-level SEM did not explicitly account for clustering, sampling weights, or robust standard errors, which may have affected the precision and statistical significance of the estimates. While the structural equation modeling approach enables the exploration of theoretically informed pathways, causal inferences remain tentative. Future research should employ longitudinal or experimental designs and multilevel or cluster-robust analytical approaches to validate these mechanisms and better account for the nested data structure.

Third, this study focused on two key psychological mediators—cognitive engagement and subject-specific efficacy—based on their theoretical relevance in social-cognitive and constructivist frameworks. However, additional mediating mechanisms, such as learning interest, self-regulated learning behaviors, emotional engagement, or classroom climate perceptions, may also play critical roles in linking instructional practices to student outcomes. Future research could explore these additional pathways to develop a more comprehensive understanding of the psychological processes involved.

Fourth, the use of publicly available TALIS data imposes certain constraints. The variables included in the analysis are limited to those collected and released by the OECD, which may restrict the ability to capture some nuanced or context-specific teaching behaviors and student responses. Moreover, although the dataset is high-quality and internationally benchmarked, it may still be subject to self-report biases, particularly in responses from teachers and students. These limitations should be considered when interpreting the findings and their broader implications.

## Data Availability

Publicly available datasets were analyzed in this study. This data can be found at: https://web-archive.oecd.org/2020-11-16/569866-GTI%20Data%20csv.zip.

## Footnotes

1 Our analysis highlights a nuanced, rather than inherently detrimental, role of classroom management. Although a negative path from classroom management to the quantity of classroom interaction was observed (*β* = −0.073, *p* < 0.001), the small coefficient cautions against any simplistic conclusion that structural order harms student learning. Instead, several alternative sociocultural and methodological explanations must be considered. First, within Shanghai’s specific classroom culture, which is heavily anchored in Confucian educational values, high behavioral compliance and teacher-directed organization are culturally normative and valued. A highly structured lesson format may reduce the absolute frequency or runtime of student-initiated talk without undermining the cognitive focus of the lesson. Second, traditional respect for teacher authority means that efficient classroom transitions and monitoring often occur through silent alignment rather than extensive dialogic negotiation. Finally, this minor negative path could stem from measurement and coding limitations within the GTI framework, where standard observation rubrics might prioritize overt behavioral indicators of interactive ‘quantity’ while under-capturing subtle or implicit cognitive engagement unique to East Asian high-stakes instructional settings.

## Appendix A: instrument

**Table:**

Variable	Components/description	Code (from GTI coding system)
Classroom management	Routines Monitoring Disruptions	Expert rating of the video VCOMP_CM1RT/CM2MN/ CM3DS
Social–emotional support	Respect Encouragement and Warmth	Expert rating of the video VCOMP_ SE1RP/ SE2EW
Quality of subject matter	Explicit Connections Explicit Patterns and Generalizations	Expert rating of the video VCOMP_QS1EC/QS2PG
Quality of classroom interaction	Questioning Explanations	Expert rating of the video VCOMP_DC2QT/DC3EP
Quantity of classroom interaction	Nature of Discourse Risk-taking Discussing Opportunities	Expert rating of the video VCOMP_DC1ND/SE3RT/ DC1DOMAX
Cognitive engagement	Actual cognitive engagement	Self-reported questionnaire data SB_USECOGACT
Subject-specific efficacy	Students’ self-efficacy in learning quadratic equations	Self-reported questionnaire data POST_GENSELFEFF
Learning outcome	Mathematics academic achievement	Mathematics academic achievement POST_TEST*

## Appendix B: descriptive statistics and correlations among study variables

**Table:**

No.	Variable	N	M	SD	1	2	3	4	5	6	7
1	Classroom Management	2,579	3.743	0.062	—						
2	Social–emotional Support	2,579	2.631	0.231	0.075***	—					
3	Quality of Subject Matter	2,579	1.974	0.222	0.037	0.396***	—				
4	Quality of Classroom Interaction	2,579	2.508	0.205	0.075***	0.565***	0.590***	—			
5	Quantity of Classroom Interaction	2,579	1.935	0.294	−0.028	0.539***	0.330***	0.485***	—		
6	Cognitive Engagement	2,579	1.699	0.258	0.282***	0.504***	0.460***	0.613***	0.533***	—	
7	Subject-specific Efficacy	2,576	3.241	0.620	0.032	0.132***	0.103***	0.123***	0.094***	0.145***	—
8	Learning Outcome	2,579	233.303	23.976	0.005	0.118***	0.100***	0.088***	0.070***	0.095***	0.360***
